# Perspective for Donor-Derived Cell-Free DNA in Antibody-Mediated Rejection After Kidney Transplantation: Defining Context of Use and Clinical Implications

**DOI:** 10.3389/ti.2024.13239

**Published:** 2024-08-12

**Authors:** Aylin Akifova, Klemens Budde, Michael Oellerich, Julia Beck, Kirsten Bornemann-Kolatzki, Ekkehard Schütz, Bilgin Osmanodja

**Affiliations:** ^1^ Department of Nephrology and Intensive Care, Charité—Universitätsmedizin Berlin, Berlin, Germany; ^2^ Department of Clinical Pharmacology, University Medical Center Göttingen, Göttingen, Germany; ^3^ Chronix Biomedical GmbH, Göttingen, Germany

**Keywords:** kidney transplantation, antibody-mediated rejection, molecular diagnostics, donor-derived cell free DNA, donor-specific antibodies (DSA)

## Abstract

Antibody-mediated rejection (AMR) is a major cause of graft failure limiting long-term graft survival after kidney transplantation. Current diagnostic strategy to detect AMR is suboptimal and requires further improvement. Previously suggested treatment regimens for AMR could not demonstrate efficacy, however novel therapeutic agents are currently under investigation. Donor-derived cell-free DNA (dd-cfDNA) is a novel non-invasive biomarker for allograft injury, that has been mainly studied in the context of rejection. Its short-half-life in circulation and injury-dependent release are its key advantages that contribute to its superior diagnostic accuracy, compared to traditional biomarkers. Moreover, previous studies showed that dd-cfDNA-release is well-linked to histological and molecular features of AMR, and thus able to reflect real-time injury. Further observations suggest that dd-cfDNA can be used as a suitable screening tool for early detection of AMR in patients with donor-specific-anti-HLA-antibodies (DSA), as well as for monitoring AMR activity after anti-rejection treatment. The weight of evidence suggests that the integration of dd-cfDNA in the graft surveillance of patients with AMR, or those suspicious of AMR (e.g., due to the presence of donor-specific anti-HLA-antibodies) has an added value and might have a positive impact on outcomes in this specific cohort.

## Introduction

Antibody-mediated rejection (AMR) is associated with inferior long-term outcomes and is a leading cause of premature graft loss after kidney transplantation [1, 2]. The complex pathogenesis behind AMR, along with its variable clinical course, provides a diagnostic and therapeutic challenge for transplant physicians today and requires novel strategies for the future [3]. The emerging era of personalized immunosuppression demands new advances in monitoring allograft health, especially for kidney transplant recipients (KTR) at high risk for AMR, e.g., sensitized recipients as well as KTR who develop *de novo* donor-specific anti-HLA antibodies (dnDSA). Standard-of-care surveillance biomarkers, such as serum creatinine and urine albumin, are not sufficient for detecting early subclinical AMR and cannot differentiate kidney allograft dysfunction due to AMR from other causes.

According to the Banff classification, serological evidence of DSA is one of the main criteria for the definite diagnosis of AMR [4]. However, despite global assay standardization [5], posttransplant timing and frequency of DSA screening is not uniformly established and there is no robust evidence for the benefit of routine monitoring of pre-formed DSA in the posttransplant course [6]. Furthermore, two recent studies showed no added value of systematic DSA screening for detection of AMR at an earlier stage [7, 8]. While development of dnDSA was predictive of graft failure, optimization of baseline immunosuppression and adherence training in the OuTSMART trial only had a limited intervention effect, such as reduction in biopsy-proven rejection in the treated group, while optimization of baseline immunosuppression and adherence training had no impact on allograft survival in this trial [8].

Besides limited treatment options for AMR, other aspects are challenging for a straightforward implementation of regular screening for dnDSA: DSA can fluctuate, become undetectable, and reappear over time, which does not necessarily reflect the immune-mediated damage or correspond with the clinical course [9, 10]. Some DSA characteristics, such as mean fluorescence intensity (MFI), IgG-subclass, or complement-binding capacity were studied to estimate the clinical impact of DSA and predict AMR outcomes, but the diagnostic value and immediate clinical utility remained uncertain [11–14].

While the first appearance of dnDSA is associated with clinical AMR in only 6.5% of patients, the majority of cases are initially uneventful without injury [10]. Therefore, it is unclear whether occurrence of DSA (i) sets the beginning of a latent alloimmune injury ultimately leading to AMR, (ii) is a first measurable sign of subclinical AMR, (iii) or simply represents a laboratory artifact without any clinical significance. Given the poor prognosis after first detection of dnDSA, this clinical dilemma requires a novel approach for better risk stratification, especially in DSA-positive KTR with stable graft function. Any strategy to prevent the progression of DSA-mediated injury requires an early detection of ongoing injury. The failure of previously investigated treatment regimens [15–17] could be partly explained by a delayed diagnosis and the presence of already irreversible chronic injury at treatment initiation.

Performing kidney allograft biopsy remains the gold standard to verify or rule out suspected rejection. Although it is a well-established low-risk procedure, it is not completely risk-free, being an invasive intervention that usually requires hospitalization and still has its limitations such as sampling errors, inter-observer variability, and the non-specificity of histological lesions [18, 19]. Another crucial aspect is the fact that biopsies triggered by the detection of DSA confirm AMR in less than 50%, whereas indication biopsies triggered by deterioration of the graft function usually describe advanced rejection stages as well as collateral damage that might further limit treatment initiation and efficacy [20–23].

Altogether, this leads to a significant diagnostic gap, and precise and early detection of subclinical AMR is an unmet medical need, that needs to be addressed by the transplant community. As current screening strategies with creatinine, urine protein, and DSA are unable to detect subclinical AMR, a combined screening strategy with an additional biomarker beyond anti-HLA-DSA may be the path forward [6, 24].

Donor-derived cell-free DNA (dd-cfDNA) is a non-invasive biomarker indicating allograft injury that has recently gained attention for the care of patients after solid organ transplantation and was deemed promising in identifying rejection with greater accuracy than traditional parameters [25, 26]. Previous research mainly focused on discriminating rejection from no rejection and demonstrated superior diagnostic performance in discriminating AMR compared to T-cell-mediated rejection (TCMR) and borderline changes [27–32]. However, it is important to keep in mind that dd-cfDNA release is an unspecific marker of graft injury, not limited to rejection phenotypes, and is also observed in microvascular inflammation in the absence of DSA (DSAnegMVI), BK-virus associated nephropathy (BKVAN), other infections and ischemia-reperfusion injury [33–36].

Given these considerations and other relevant aspects such as availability, cost-efficiency, and feasibility, a broad and unselected use of dd-cfDNA may not be the ideal approach to advocate for the integration of this biomarker into clinical routine [37]. Instead, defining a suitable context of use is recommended, where dd-cfDNA could serve a well-defined population (such as DSA-positive patients) as a potentially useful biomarker to facilitate early detection of AMR and to guide treatment for improved outcomes in AMR [24, 37]. Hence, evidence will be reviewed on whether dd-cfDNA surveillance has the potential to be practice-changing in the contemporary management of AMR in KTR with DSA.

## Methods of Quantification and Availability

To date, several methods of detection have been established to assess dd-cfDNA in transplant recipients’ blood. All detection methods rely on genetic differences between donor and recipient DNA and share the advantage that no separate genotyping of the donor is needed. Available tests are based on highly abundant genetic polymorphism, such as single nucleotide polymorphisms (SNPs), insertion/deletion polymorphisms (indels) or copy number variations to distinguish between graft-derived and recipient DNA using new generation sequencing (NGS)-based and polymerase chain reaction (PCR)-based assays [38–43].

Nevertheless, it is important to acknowledge that there are some differences between the quantification methods that should be addressed before adopting dd-cfDNA as a diagnostic tool to guide clinical decisions. Unlike NGS techniques, quantitative PCR-based methods enable the assessment of both absolute and relative levels of dd-cfDNA [43]. Several studies showed that relative quantification is more likely to be error prone, as it can be influenced by variations in the total recipient cfDNA by non-physiological increases in cell turnover, such as cases of severe infection or malignancy where it should be interpreted cautiously. Since cfDNA is primarily released by recipient leucocytes, fluctuations in leucocyte numbers can impact absolute recipient cfDNA and consequently relative content of dd-cfDNA. Absolute values of dd-cfDNA can be affected by pre-analytical changes, such as variation in DNA extraction efficiency and cfDNA degradation in the bloodstream [25]. For values around a predefined threshold, this might lead to false-negative or false positive results, misinterpretation and even triggering unnecessary biopsies.

To overcome these limitations, it has been proposed to add absolute quantification, which demonstrates a greater diagnostic accuracy compared to the dd-cfDNA% fraction [44, 45]. Therefore, some authors support a combination of both relative and absolute quantification in the context of the total cfDNA concentration, which might enhance comprehensiveness in the decision-making process [44–47].

Another practical aspect for clinical decision-making is the turnaround time, defined as the time from blood draw to result. In some cases, it can take up to a week if the sample is shipped to a central laboratory, which not only represents a relevant logistical burden but also has consequences for its clinical implementation. Besides, when comparing the different quantification methods, sequencing in NGS processing alone can take up to 30 h and therefore is more labor-intensive, compared to the droplet digital PCR (ddPCR) approach, which can offer same-day results and is easier in set-up. From a clinical perspective, it would be advantageous if the dd-cfDNA processing could be conducted at a local laboratory with a short turnaround time, which would directly lead to faster treatment decisions while also increasing availability of the test [43].

## Correlation of dd-cfDNA With Histological and Molecular AMR

Overall, most studies demonstrated good-to-very-good test characteristics of dd-cfDNA (sensitivity, specificity, positive predictive value (PPV), negative predictive value (NPV)) for detecting rejection with best performance in discriminating AMR and mixed rejection from TCMR and borderline [26–32]. However, positive predictive value (PPV) and negative predictive value (NPV) varies depending on the rejection prevalence in the screening cohort [48]. Most studies reported remarkably high NPV for AMR, which indicates that dd-cfDNA can be used to reliably rule out underlying AMR in DSA-positive KTR with a quiescent clinical course [24].

To gain a more granular understanding of dd-cfDNA release, several studies aimed to examine the association between dd-cfDNA and histological and molecular rejection features in dd-cfDNA-paired kidney allograft biopsies. This was first explored in a small cohort (n = 37) by Zhang et al. who did not demonstrate any statistical differences between the different Banff categories and within Banff lesion scores [29]. In contrast, a subsequent analysis from a larger cohort (n = 106) with a higher prevalence of rejection found a significant correlation between dd-cfDNA and glomerulitis (g) and intimal arteritis (v), respectively, and also observed higher dd-cfDNA levels with greater Banff lesion severity [49]. Furthermore, they described an association of dd-cfDNA with microvascular inflammation score (MVI>2) and severe interstitial inflammation (i3) but not with chronic features of AMR, such as the cg (glomerular basement membrane double contours), interstitial fibrosis (ci), tubular atrophy (ct), and vascular fibrous intimal thickening (cv), which was partially confirmed in subsequent studies [42, 50–52].

Notably, the majority of the following studies demonstrated a distinct correlation of dd-cfDNA with the Banff peritubular capillaritis (ptc) score. This was seen in AMR but also in other circumstances of ptc, irrespective of the underlying diagnosis [50–52]. Interestingly, Whitlam et al. correlated both relative and absolute dd-cfDNA with the Banff scores and found that an increase in absolute dd-cfDNA was linked to active AMR features, such as g, ptc and C4d-staining, whereas dd-cfDNA fraction was also associated with other lesions, but was less specific for AMR and rejection in general [42].

The prospective multicenter Trifecta-Kidney study (NCT04239703) was conducted to calibrate dd-cfDNA against conventional histology and the molecular archetypes in the Molecular Microscope Diagnostic system (MMDx) according to rejection stage, severity, and activity, as previously introduced by the MMDx study group [53, 54]. The study extensively investigated anti-HLA-antibodies and histology, follow-up data, and clinicians’ feedback to explore the relationship between these findings in this multi-layered diagnostic approach. Results from 300 matched biopsies were analyzed to correlate dd-cfDNA with histologic and molecular features associated with AMR and overall rejection. Random forest analysis verified ptc as the most important variable associated with increased dd-cfDNA, followed by the much weaker g- and v-scores. Among the molecular variables, the molecular ptc-lesion classifier (ptc_Prob_) was the top predictor of elevated dd-cfDNA, followed by the all-rejection (Rej_Prob_) classifier and the ABMR probability classifier (ABMR_Prob_) [52]. Overall, the prediction of increased dd-cfDNA was more accurate with the molecular variables than with the histological variables for AMR, which was confirmed in a real-world prospective study by Gupta et al. [55].

Another key aspect is the relationship between dd-cfDNA and AMR archetypes in MMDx, e.g., dd-cfDNA being the highest in fully developed AMR (FABMR) with a tendency to be lower in late AMR (LABMR), which is usually less active, as described previously [54]. These insights strongly underscore the hypothesis that dd-cfDNA is linked to the severity of the injury as reflected by the molecular AMR activity. Under this assumption, dd-cfDNA could be an easily obtained non-invasive biomarker that potentially reflects AMR activity and outperforms standard clinical and conventional biomarkers such as DSA [56, 57].

In summary, current evidence strongly suggests that high dd-cfDNA values are associated – but not exclusively - with microvascular inflammation (MVI) in the allograft, which is of high diagnostic significance, as the MVI lesion is a hallmark of AMR, although it is seen in other circumstances as well. Given the relevant discrepancy between histology and MMDx, with a tendency for AMR being underdiagnosed in conventional microscopy [58], high dd-cfDNA levels can provide additional evidence for AMR or make AMR less likely in cases with DSA and ambiguous or insufficient histology.

## dd-cfDNA for Early Diagnosis of AMR

Early diagnosis of therapy-requiring conditions in general and AMR in particular, is essential and should also be recognized as an important determinant for improved patient and graft survival after kidney transplantation [59, 60]. In the specific context of AMR, early detection is crucial since biopsies presenting with chronic AMR changes at initial diagnosis are associated with more adverse outcomes and could rapidly progress to graft loss [22, 23]. Given its key features, like the short half-life in circulation and injury-dependent release, dd-cfDNA can signalize the onset of antibody-mediated damage much earlier and preceding clinical deterioration in KTR with stable graft function. In the advent of novel therapeutic agents, such as CD38 targeting monoclonal antibodies, the early detection of subclinical AMR may become increasingly important [61].

This is supported by findings of the multicenter, observational ADMIRAL study (NCT04566055), which was the largest study to prospectively follow up KTR with dd-cfDNA surveillance as part of the routine monitoring. In this cohort, high dd-cfDNA values were observed in a significant proportion of AMR biopsies of KTR with no major impairment in kidney allograft function, as defined by a decline in eGFR or proteinuria [62]. Another retrospective observational study, where longitudinal assessment pre-biopsy was available, also showed that the determination of elevated dd-cfDNA levels could have led to an earlier detection of AMR [63].

**FIGURE 1:**
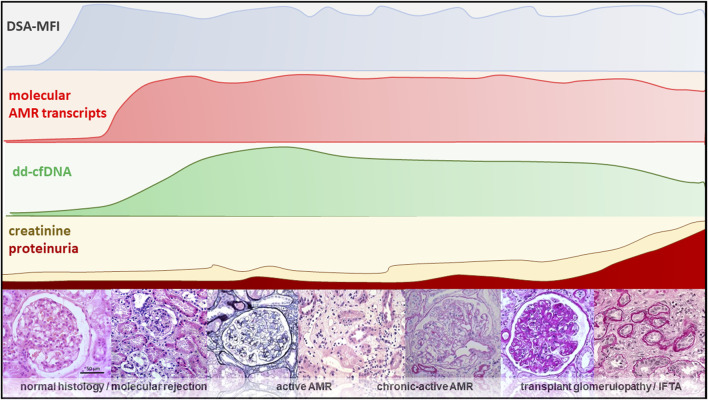
An overview of different diagnostic modalities showing the relationship between dd-cfDNA and the molecular, clinical and histological progression within the natural course of AMR [65–69].

These observations were supported by a recently completed, single-center, diagnostic randomized clinical trial (NCT04897438). In this trial, patients with a functioning kidney graft and dnDSA without evidence of AMR, were assigned to either intervention (dd-cfDNA-guided biopsy) or a control group (clinician-guided-biopsy), and dd-cfDNA was longitudinally assessed in both groups over 1 year. Increase over the predefined absolute threshold in the intervention group, indicated a diagnostic biopsy, regardless of kidney function. The primary endpoint “time to AMR-diagnosis” was met by a significant 9-month earlier AMR diagnosis in the intervention group, compared to the control group [64]. Again, dd-cfDNA had very good test characteristics (sensitivity 83%, specifity 79%, PPV 0.75, NPV 0.85) in dnDSA-positive KTR, extending previous observations [27–32]. This is the first prospective randomized trial that provides evidence for the potential benefit of dd-cfDNA monitoring in KTR with dnDSA. The data suggest that, the additional determination of dd-cfDNA can reliably identify AMR in an early and potentially reversible stage of rejection and enable timely therapeutic intervention (Figure 1).

**FIGURE 2:**
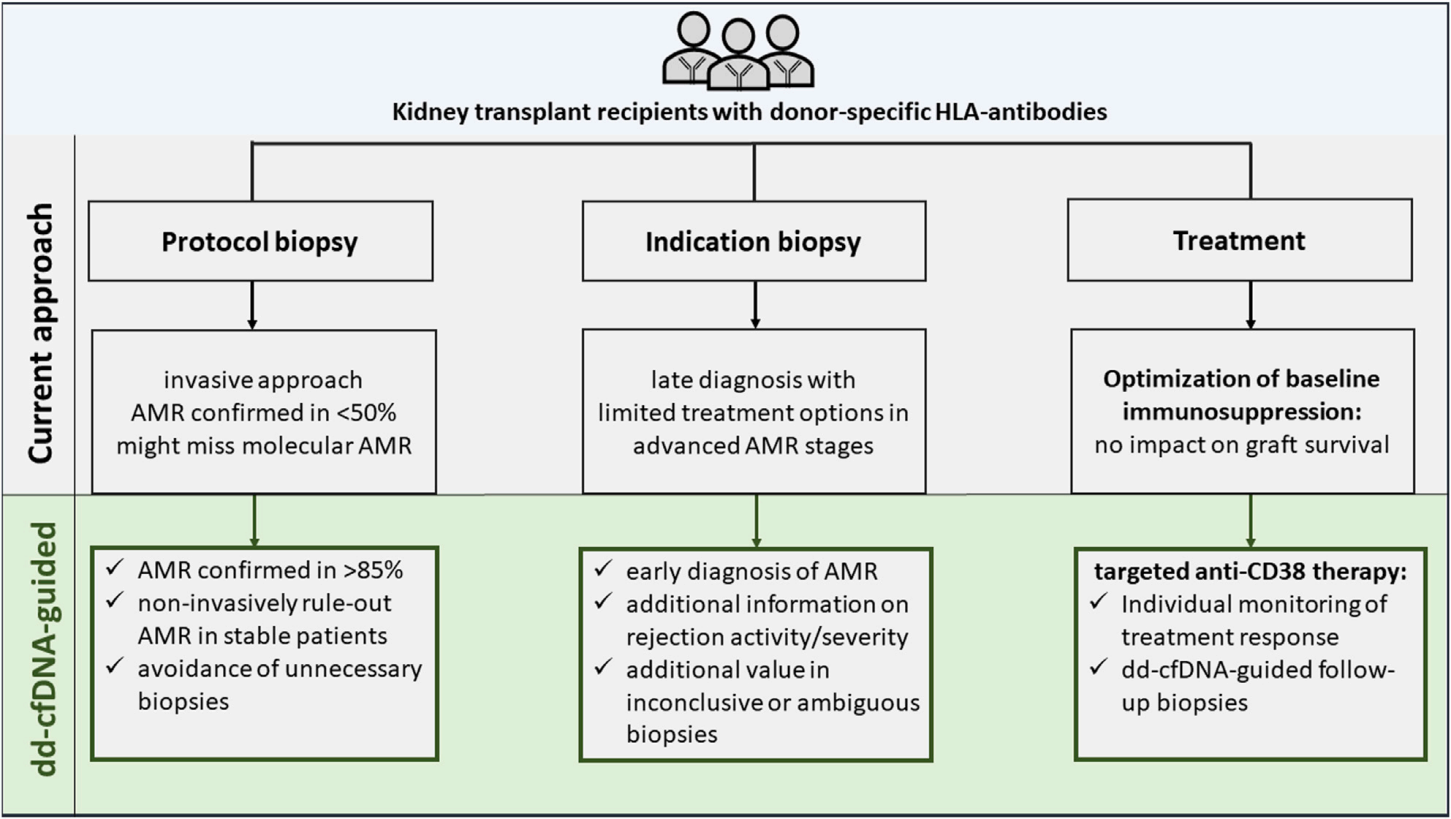
Current versus donor-derived cell-free DNA-guided diagnostic and therapeutic approach in kidney transplant recipients with donor-specific antibodies.

Contrary to previous approaches, dd-cfDNA was assessed in a well-defined cohort of KTR with DSA, thereby increasing the pre-test probability and, explaining the favourable test characteristics. Within this context of use, a dd-cfDNA-guided biopsy would dramatically reduce unnecessary biopsies by around 50% compared to a general protocol biopsy approach in DSA positive KTR, where only 50% of biopsies reveal AMR (Figure 2) [20, 21].

Another randomized controlled experiment addressed the clinicians perspective, namely, how the availability of complementary dd-cfDNA results will affect the decision-making process. Interestingly, the study showed that nephrologists aware of the dd-cfDNA results were more likely to correctly diagnose early and subclinical graft rejection, refer to a transplant center, or appropriately change treatment compared to nephrologists without dd-cfDNA access [70]. Future multicenter prospective outcome studies are warranted to deliver a greater power of these promising findings.

## Therapeutic Challenge of AMR and the Potential of dd-cfDNA to Monitor Treatment Success

Along with the continuous efforts to develop therapy solutions for AMR, a suitable tool to assist in validating the success of investigational or available treatment is also desirable. Traditional parameters may not be ideal for guiding therapeutic response due to their non-specificity and inability to reflect resolving injury. As a result, follow-up biopsies are required to assess whether and to what extent applied anti-rejection treatment was efficacious.

Unlike TCMR or borderline rejection, AMR remains a major therapeutic challenge [3], and without approved treatment, timely diagnosis of AMR may not be ultimately helpful. Better classification and ability to include early subclinical AMR into treatment trials may contribute to successful drug discovery [3]. Although different agents have been investigated in the attempt to target potentially pathogenic pathways, no substance or multimodal regimen led to an evident success so far [15–17, 71]. Several studies suggested some stabilization of the graft function, and novel therapy concepts are currently under investigation in randomized-controlled trials (NCT04561986, NCT05156710, and NCT05021484) [61, 72, 73]. Recently, a phase-3 trial was terminated due to lack of efficacy in the interim analysis (NCT03744910).

There is some anecdotal evidence for the use of dd-cfDNA after anti-rejection treatment. Hinojosa et al. first suggested that dd-cfDNA might confirm real-time response to treatment after acute rejection [74]. This was confirmed in a larger cohort consisting of the rejecting biopsies identified in the DART study, where dd-cfDNA decrease was observed in both TCMR and AMR biopsies with longitudinal surveillance after treatment [75]. A similar trend towards decreasing dd-cfDNA values in some patients was reported in two studies that performed dd-cfDNA follow-up monitoring after rejection, which were mostly interpreted as therapy response [51, 55]. Similarly, a single-center report described dd-cfDNA to assess treatment response of the monoclonal anti-IL-6-receptor antibody tocilizumab in patients with AMR and observed a significant decrease in dd-cfDNA, potentially reflecting resolving injury, but only a nonsignificant trend in proteinuria reduction [76]. In contrast, Mayer et al. could not confirm changes in dd-cfDNA values after treatment with anti-IL6 antibody clazakizumab in patients with AMR, suggesting ongoing injury [77].

Advances in unveiling the pathogenesis of AMR focused on the role of natural killer-cells (NK-cells) in AMR and microvascular inflammation in kidney allografts [78]. Accordingly, anti-CD38 antibodies are becoming interesting candidates to target antibody-producing plasma cells as well as NK-cells that are described to be key injury mediators in AMR [78–81]. Recently, results from the first randomized-controlled phase-2 trial (NCT05021484) investigating the novel anti-CD38 agent felzartamab (fully human IgG1 monoclonal anti-CD38 antibody) in KTR with late AMR suggest the potential efficacy of this targeted approach [72, 82].

Together with resolution of AMR activity in felzartamab-treated patients, a rapid normalization of dd-cfDNA was seen. Again, dd-cfDNA levels were highly correlated with AMR activity in histology and molecular AMR scores. Six months after treatment discontinuation, recurrence of AMR activity was seen in some patients together with increasing dd-cfDNA levels and molecular AMR scores. Together, these data further support the hypothesis that dd-cfDNA is a reliable biomarker, closely related to AMR activity, that could serve as a companion diagnostic to guide treatment response in patients with AMR (Figure 2) [82].

The full utility of dd-cfDNA as a companion biomarker to guide anti-rejection therapy is to be determined in future prospective multicentric trials. It might help monitor individual treatment response, avoid repeat biopsies to control treatment response or perform such with greater precision through dd-cfDNA-triggered indication.

## Conclusion

Dd-cfDNA is a sensitive biomarker of ongoing cellular injury in the transplanted kidney. Although not specific for AMR, dd-cfDNA has a promising potential to add value for the detection, diagnosis, and monitoring of AMR after kidney transplantation. Most importantly, the context of use must be well defined, as for any other test. It cannot replace DSA screening, but in patients with DSA, it provides additional information that enables earlier detection of AMR and may help to avoid unnecessary biopsies in patients with low dd-cfDNA values due to its high negative predictive value. In addition, dd-cfDNA may help to better judge the severity of ongoing AMR, although more studies are needed to fully establish the relationship of dd-cfDNA with outcomes in AMR patients. The correlation between dd-cfDNA, microvascular inflammation in histology and molecular AMR may also translate into a better understanding of the evolution of subclinical AMR. Finally, evidence is evolving that dd-cfDNA may be a valuable non-invasive tool for monitoring ongoing AMR activity and potential treatment effects.

We believe that dd-cfDNA has an added clinical value for the timely diagnosis of AMR in DSA-positive patients and may also be useful for monitoring AMR treatment response, as the integration of this biomarker into clinical care of DSA-positive patients would reduce the need for biopsies due to high positive and negative predictive values. However there are still some limitations and knowledge gaps that deserve consideration and require additional research, such as the biological background of release, optimal frequency of dd-cfDNA testing, comparability and cost of different assays as well as cost-effectiveness of different approaches [24, 37]. Within the context of DSA-positive patients more data are needed for an optimal clinical implementation and ideally future prospective-randomized clinical trials with robust endpoints and sufficient follow-up will provide more evidence on the clinical impact of complementary dd-cfDNA monitoring on the clinical course of patients with DSA and AMR.

Other aspects that deserve attention are the use in other indications such as DSA-negative-MVI, and BKVAN. Beyond this, other biomarker candidates, such as peripheral blood gene-expression tests, urinary chemokines (e.g., interferon gamma (IFNG) dependent, CXCL9 and CXCL10) and the Torque-Teno-virus (TTV), are also being studied to further improve graft surveillance. However, only a few reports specifically addressed their utility in patients with AMR. Contrary to dd-cfDNA, preliminary results for patients with AMR are conflicting and evidence limited. Additional aspects that limit their transition into clinical routine are reproducibility, assay standardization and availability. More focused research or strategies with a combined approach might help to better define their role for monitoring patients with DSA or AMR in the future [24, 77, 83–85].

In conclusion, dd-cfDNA is currently the best available, non-invasive biomarker, beyond anti-HLA-DSA, to identify patients with AMR. The current evidence suggests that the integration of dd-cfDNA in the graft surveillance of patients with AMR, or those suspicious of AMR (e.g., due to the presence of DSA) has additional value and may help to improve outcomes in this specific cohort. dd-cfDNA could be helpful in personalized post-transplantation therapy with the potential to reduce premature graft loss.

## Abbreviations

AMR, antibody-mediated rejection; cfDNA, cell-free DNA; ci, interstitial fibrosis; cg, glomerular basement membrane double contours; CNI, calcineurin inhibitor; ct, tubular atrophy; cv, vascular fibrous intimal thickening; dd-cfDNA, donor-derived cell-free DNA; ddPCR, droplet digital PCR; dnDSA, *de novo* donor-specific anti-human leukocyte antigen antibodies; DSA, donor-specific anti-human leukocyte antigen antibodies; eGFR, estimated glomerular filtration rate; g, glomerulitis; HLA, human leukocyte antigen; i, interstitial inflammation; IgG, immunoglobulin G; KTR, kidney transplant recipient; MFI, mean fluorescence intensity; MMDx, Molecular Microscope Diagnostic System; MVI, microvascular inflammation; NGS, next-generation sequencing; NPV, negative predictive value; PPV, positive predictive value; ptc, peritubular capillaritis; SNP, single nucleotide polymorphisms; TCMR, T-cell mediated rejection; uACR, urine albumin-creatinine ratio; v, intimal arteritis.
